# Severe Generalized Epidermolysis Bullosa Simplex in Two Hong Kong Children due to *De Novo* Variants in *KRT14* and *KRT5*

**DOI:** 10.1155/2020/4206348

**Published:** 2020-04-17

**Authors:** Shuk Ching Chong, Kam Lun Hon, Fernando Scaglia, Chung Mo Chow, Yu Ming Fu, Tor Wo Chiu, Alexander K. C. Leung

**Affiliations:** ^1^Department of Paediatrics, The Chinese University of Hong Kong, Prince of Wales Hospital, Shatin, Hong Kong; ^2^The Chinese University of Hong Kong, Baylor College of Medicine Joint Center for Medical Genetics, Prince of Wales Hospital, Shatin, Hong Kong; ^3^Department of Molecular and Human Genetics, Baylor College of Medicine, Houston, Texas, USA; ^4^Texas Children's Hospital, Houston, Texas, USA; ^5^Department of Paediatrics and Adolescent Medicine, Princess Margaret Hospital, Kwai Chung, Hong Kong; ^6^Division of Plastic Reconstructive and Aesthetic Surgery, The Chinese University of Hong Kong, Prince of Wales Hospital, Shatin, Hong Kong; ^7^Department of Pediatrics, The University of Calgary and the Alberta Children's Hospital, Calgary, Alberta, Canada

## Abstract

We report two Hong Kong children with severe generalized epidermolysis bullosa simplex (EBS), the most severe form of EBS, without a family history of EBS. EBS is a rare genodermatosis usually inherited in an autosomal dominant fashion although rare autosomal recessive cases have been reported. Genetic studies in these patients showed that the first case was due to a novel *de novo* heterozygous variant, c.377T>G (NM_000526.5 (c.377T>G, p.Leu126Arg)) in the *KRT14* gene and the second case was due to a rare *de novo* heterozygous variant c.527A>G (NM_000424.4, c.527A>G, p.Asn176Ser) in the *KRT5* gene. To our knowledge, the c.377T>G variant in the *KRT14* gene has not been previously reported, and the c.527A>G variant in the *KRT5* gene is a rare cause of severe generalized EBS. In severe generalized EBS, infants exhibit severe symptoms at the onset; however, they tend to improve with time. A precise genetic diagnosis in these two cases aided in counseling the families concerning the prognosis in their affected children and the recurrence risk for future pregnancies.

## 1. Introduction

Epidermolysis bullosa (EB) is a heterogeneous group of rare inherited connective tissue disorders characterized by marked fragility of epithelial tissues with prototypic blistering, erosions, and nonhealing ulcers following minimal rubbing or frictional trauma [1–10]. EB is classified into four major categories, each with many subtypes based on the precise location at which separation or blistering occurs, namely, epidermolysis bullosa simplex (EBS; intraepidermal skin separation), epidermolysis bullosa junctional (EBJ; skin separation in lamina lucida or central basement membrane zone (BMZ)), dystrophic epidermolysis bullosa or epidermolysis bullosa dystrophica (EBD; sublamina densa BMZ separation), and Kindler syndrome (multiple cleavage planes) [2–4, 6, 8, 11–14]. The fundamental pathology of EB lies on the increase in collagenase activity, leading to collagen degeneration and hence splitting of various epidermal layers or at the transition between epidermis and dermis [5, 15]. EBS is the most common type of EB, accounting for 75 to 85% of cases of EB in the Western world [16]. EBS is usually caused by pathogenic variants in the keratin genes (*KRT5* and *KRT14*) with resultant formation of a cleavage plane at the level of the basal keratinocytes [17]. Localized EBS (formerly known as Weber–Cockayne EBS), usually associated with little or no extracutaneous involvement, is the mildest and most common form of EBS. Nail dystrophy is rare and generally mild. Severe generalized EBS (formerly known as Dowling–Meara EBS) is the most severe form of EBS and presents with widespread friction-induced blistering at birth. Involvement of the oral mucosa and nail dystrophy are common. Generalized intermediate EBS (formerly known as Koebner EBS) may present at birth with blistering and possibly with milder clinical courses [18–21].

A retrospective review of EB cases diagnosed and evaluated at the Department of Pediatrics at Prince of Wales Hospital in Hong Kong was conducted [22]. There were only two cases of congenital EBS diagnosed over the past 20 years (1999 to 2019). Their demographic details, clinical presentation, histopathology findings, and genetics findings were reviewed. Genetic testing included a next generation sequencing (NGS) EB panel and Sanger sequencing technologies to cover the full coding regions and ∼10 bp of noncoding DNA flanking region of each exon of the genes related to EB. Genomic DNA was extracted from the patient and parents' blood specimens. For NGS, patients' DNA was captured, and then sequenced using Illumina's Reversible Dye Terminator (RDT) platform. Sanger sequencing was used for parental sample testing. Ethics approval was obtained from the NTEC-Chinese University of Hong Kong Ethics Committee to review these cases, and consent was obtained from both families.

In this report, we describe two Hong Kong children with severe generalized EBS. The first case was due to a novel *de novo* heterozygous c.377T>G (NM_000526.5 (c.377T>G, p.Leu126Arg)) variant in the *KRT14* gene, and the second case was due to a rare *de novo* heterozygous c.527A>G (NM_000424.4, c.527A>G, p.Asn176Ser) variant in the *KRT5* gene. To our knowledge, the c.377T>G variant in the *KRT14* gene has not been reported previously, and the c.527A>G variant in the *KRT5* gene is a rare cause of generalized severe EBS.

## 2. Case Series

### 2.1. Case 1

A female neonate was born to nonconsanguineous Southern Chinese parents at term following an uncomplicated pregnancy and normal spontaneous vaginal delivery. Her birth weight was 2.455  kg. She was noted to have extensive bullous lesions over the whole body and blisters in the buccal mucosa at birth. Dystrophic nail changes in the fingers and toes were also noted. There was no family history of bullous disease. A skin biopsy was performed. Attachment of the basement membrane to the blister roof could not be clearly determined by light microscopy. Electron microscopy revealed EBS characterized by a diffuse cytolysis in basal cells with intraepidermal cleavage.

NGS detected a novel *de novo* heterozygous c.377T>G variant in the *KRT14* gene that predicted to result in the amino acid substitution p.Leu126Arg (NM_000526.5 (c.377T>G, p.Leu126Arg)). No copy number variants were found in *KRT14*. She also had a novel *ITGB4* variant (NM_000213.5 (c.3554A>G, p.Asn1185Ser)). Clinically, this patient did not have pyloric atresia. The asymptomatic father did not carry the same variants on *ITGB4* nor *KRT14* gene. Both her asymptomatic mother and elder brother carried the same *ITGB4* variant. This patient had recurrent crops of generalized bullous formation and significant failure to thrive. She had poor weight gain and frequent infections during the first few months of life. The condition of this patient was still very severe at follow-up at 9 months of age.

### 2.2. Case 2

**Figure 1:**
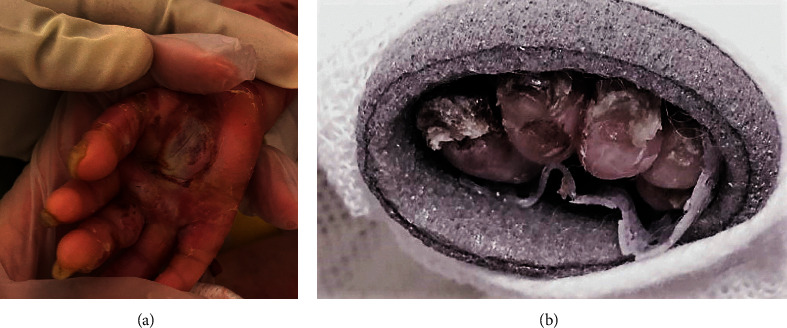
(a) Severely dystrophic fingernails of right hand and (b) toenails of the left foot wrapped in multiple layers of protective dressings.

**Figure 2:**
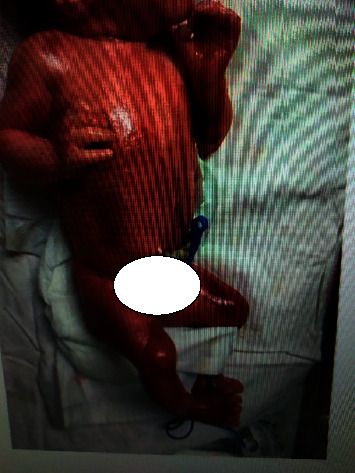
Severe generalized EBS with denuded skin following rupture of bullae involving the face, torso, and the limbs.

A female neonate was born at term following an uncomplicated pregnancy and normal vaginal delivery with a birth weight of 2.3  kg. Parents were healthy nonconsanguineous Southern Chinese. She had extensive bullae over the whole body from the skull to the soles at birth. There were blisters present in the oral mucous membranes. Dystrophic nail changes in the fingers and toes were also noted (Figure 1). There was no family history of bullous disease. Genetic testing confirmed a *de novo* heterozygous variant in *KRT5*, c.527A>G (NM_000424.4, c.527A>G, p.Asn176Ser). No copy number variants were found in *KRT5*. The patient had poor weight gain and recurrent bacterial infections which required treatment with antibiotics. New bullae continued to develop all over her body over the first few months of life (Figure 2). The function of her joints was not affected. With the molecular diagnosis, no skin biopsy was obtained from this patient. She was treated with special enriched milk to increase the caloric intake and supplemented with trace elements. The skin and nails were still severely affected in the first year of life, and the child received intensive care for the first 9 months of life.

## 3. Discussion

Severe generalized EBS is devastating both to patients and their families. Obstetricians and pediatricians must be familiar with the mode of inheritance, age-related morbidity, and mortality associated with this rare but severe disease in order to provide timely counseling on the natural history of the disease, recurrence risk, and reproductive options to the families. Histopathology and molecular studies play an important role in prognostication and counseling. NGS detected that the first patient had a *de novo* heterozygous novel c.377T>G variant in the *KRT14* gene (NM_000526.5 (c.377T>G, p.Leu126Arg)), which was predicted to result in the amino acid substitution p.Leu126Arg. This *KRT14* variant has not been reported in the literature and is not found in ExAC or 1000 genomes. The *in silico* prediction for this variant by SIFT and Polyphen-2 is damaging, and the amino acid residue is highly conserved across species. This variant is also predicted to be deleterious when analyzed by the Mutation Taster software (http://www.mutationtaster.org/). No copy number variants were found in the *KRT14* gene. She also had an *ITGB4* variant (NM_000213.5 (c.3554A>G, p.Asn1185Ser)), which has not been reported in the literature to date, and it was predicted to be of uncertain clinical significance according to American College of Medical Genetics and Genomics (ACMG) guidelines. *ITGB4* encodes for the hemidesmosomal protein integrin *β*4. Pathogenic variants in *ITGB4* may cause the rare subtype of EB with pyloric atresia (EB-PA). This patient did not exhibit pyloric atresia. Both her asymptomatic mother and elder brother carried the same *ITGB4* variant. These findings help narrow down the variant in *KRT14* variant as responsible for her EBS. Genetic information of the first case was especially relevant for counseling because two EB gene variants were present, one responsible for EBS while the other was deemed not relevant in this case. Precise genetic diagnosis is highly relevant for counseling in EBS, especially in a patient without a skin biopsy as is illustrated in the second case or if the skin biopsy result is ambiguous or inconclusive. *KRT5* c.527A>G (p.Asn176Ser) is a rare variant in individuals with sporadic EBS [23]. The prognosis of *KRT5* variants has been reported among Chinese and other Asian patients [23].

EBS is almost always inherited in an autosomal dominant fashion, although rare autosomal recessive forms have been reported [24]. *De novo* pathogenic variants in *KRT14* and *KRT5* genes account for the occurrence of severe generalized EBS in these two patients in the absence of a family history of EBS. One limitation of this study is that no screening was performed in these two cases for possible somatic or germline mosaicism in their parents. However, although somatic and germline mosaicism have been found to be the underlying cause in some seemingly sporadic cases thought to be caused by *de novo* pathogenic variants [25], another study has reported a high rate of 37% for *de novo* pathogenic variants in *KRT14* and *KRT5* [26]. The cause for the high percentage of *de novo* variants is not entirely clear, but highly mutable CpG dinucleotides have been found in some codons more frequently affected by these *de novo* variants in multiple families [26].

The genetics of EBS have been reported in Korean, Japanese, and Chinese patients, but not in patients from Hong Kong (Table 1) [18, 20, 28, 29]. The current report expands the molecular spectrum of EBS. Knowledge of the exact genetics of EBS helped in counseling the families regarding the prognosis of their affected children and recurrence risk for future pregnancies.

Unlike EBD and EBJ, EBS is usually a milder disease and not associated with high mortality [30]. Our two patients had severe generalized EBS based on the onset of the disease at birth, disseminated friction or trauma-induced blistering, involvement of oral mucosa, and presence of nail dystrophy. Despite these findings, symptoms observed in severe generalized EBS tend to improve with time [30]. The main causes of early morbidity and mortality in severe generalized EB are septicemia, malnutrition, and electrolyte disturbances [1–4]. Hence, skin care and nutrition support must be meticulous [31]. Malnutrition can be attributed to recurrent mucosal lesions, feeding difficulties, high energy consumption from accelerated skin turnover, transcutaneous loss of nutrients, and catabolic state from recurrent infections [22, 31]. It is therefore important to involve dietitians to prepare easy-to-consume recipes, identify high-caloric and protein-fortified foods and beverages to replace protein lost in draining blisters, suggest vitamin and mineral nutritional supplements, and recommend dietary adjustments to prevent gastrointestinal problems, such as constipation, diarrhea, or painful defecation [4, 11, 12]. During hospitalization, the importance of adequate nutritional intake should be reinforced. When indicated, the option of gastrostomy should be discussed with the patient and his/her family for those patients who remain cachectic despite conservative measures.

## 4. Conclusion

Herein, we report two children with severe generalized EBS in Hong Kong. Severe generalized EBS is an inherited blistering skin disease associated with significant morbidity and mortality, and the prognosis is better with the autosomal dominant inherited or *de novo* EBS cases than in those with the rare autosomal recessive inherited EBS. The first case was due to a novel *de novo* heterozygous variant c.377T>G in *KRT14*, and the second case was due to a rare *de novo* heterozygous variant with c.527A>G in the *KRT5* gene. These molecular findings corroborate the elevated rate of seemingly *de novo* variants in *KRT5* and *KRT14* found in previous studies. Exact genetic diagnosis of severe generalized EBS aided in counseling the families concerning the prognosis of this disease in their affected children and the recurrence risk for future pregnancies. It would be most useful to establish a registry for EB in Hong Kong to evaluate the natural history of these disorders in order to facilitate patient management via a multidisciplinary team approach and facilitate novel therapeutic approaches such as gene therapy trials in the upcoming future.

## Figures and Tables

**Table 1:** Genetics of Congenital EBS in selected Asian reports.

Ethnicity	Genetics [27]	Year of publication (reference number)
Southern Chinese (Hong Kong) *n* = 2	*KRT14* (NM_000526.4 (c.377T>G, p.Leu126Arg)) *KRT5* (NM_000424.3 (c.527A>G, p.Asn176Ser))	Present series

Japanese, *n* = 16	*KRT 5* *KRT14*	2013 [18]

Chinese, *n* = 1	*KRT5*	2016 [20]

Korean, *n* = 15	*KRT5* (p.Val143Phe, p.Arg265Pro, p.Cys479X, p.Asn177del, and p.Glu477Lys) *KRT14* (p.Arg125Leu, p.Leu401Pro, and p.Arg125His)	2010 [28]

Chinese, *n* = 2 pedigrees	*KRT5* (a heterozygous T>A transition at nucleotide 1730, changing phenylalanine (Phe) to tyrosine (Tyr) at position 577) *KRT5* (two recurrent mutations c.1649delG (p.Gly550AlafsX77) and c.508G>(p.Glu170Lys in Chinese patients with mottled pigmentation EBS and localized EBS, respectively)	2009 [29]
